# Influence of the COVID-19 Pandemic on Parenting Stress Across Asian Countries: A Cross-National Study

**DOI:** 10.3389/fpsyg.2021.782298

**Published:** 2021-12-21

**Authors:** Sawa Kurata, Daiki Hiraoka, Aida Syarinaz Ahmad Adlan, Subhashini Jayanath, Norhamizan Hamzah, Aishah Ahmad-Fauzi, Takashi X. Fujisawa, Shota Nishitani, Akemi Tomoda

**Affiliations:** ^1^Research Center for Child Mental Development, University of Fukui, Fukui, Japan; ^2^Division of Developmental Higher Brain Functions, United Graduate School of Child Development, Osaka University, Kanazawa University, Hamamatsu University School of Medicine, Chiba University, and University of Fukui, Osaka, Japan; ^3^Department of Child and Adolescent Psychological Medicine, University of Fukui Hospital, Fukui, Japan; ^4^Japan Society for the Promotion of Science, Tokyo, Japan; ^5^Department of Psychological Medicine, Faculty of Medicine, University of Malaya, Kuala Lumpur, Malaysia; ^6^Department of Pediatrics, University of Malaya, Kuala Lumpur, Malaysia; ^7^Department of Rehabilitation Medicine, University of Malaya, Kuala Lumpur, Malaysia; ^8^Life Science Innovation Center, Faculty of Medical Sciences, University of Fukui, Fukui, Japan

**Keywords:** Coronavirus Anxiety Scale (CAS), Parenting Stress Index (PSI), school closure, parenting stress, COVID-19, Adverse Childhood Experiences (ACE)

## Abstract

**Background:** In a previous study, we demonstrated that the accumulation of parenting stress during prolonged school closures and restrictions on daily activities due to the COVID-19 pandemic in Japan indicates the need for mental health intervention for parents at higher risk of parenting stress. However, few studies have focused on parenting stress in other Asian countries, although they have experienced higher numbers of infections. The aim of the present study was to investigate whether parenting stress among caregivers increased across Asia due to school closures and restrictions on activities during the COVID-19 pandemic and to examine whether there were any country-specific, cross-country, or cross-regional risk factors for increased parenting stress.

**Methods:** We conducted an online survey immediately after the number of new cases in India significantly increased (September–November 2020). We measured parenting stress, anxiety, and fear associated with the COVID-19 crisis, as evaluated by the Parenting Stress Index, Short-Form (PSI-SF), and the Coronavirus Anxiety Scale (CAS), across three Asian countries—India (*n* = 142), Malaysia (*n* = 69), and Japan (*n* = 182)—in addition to the United States (*n* = 203). We also investigated whether respondents had adverse childhood experiences (ACE) as a risk factor for parenting stress.

**Results:** For all countries, we found significant increases in participants’ current parenting stress levels, compared to what they recalled regarding their lives before COVID-19-related restrictions and school closures were enacted. Textual analysis qualitatively identified common terms related to parenting stress across all countries. We also found a statistical model that indicated ACE in parents was a critical risk factor for higher parenting stress *via* increasing anxiety and fear related to the pandemic.

**Conclusion:** These results indicate the need to improve the mental health of caregivers who are at risk for higher levels of parenting stress during the COVID-19 pandemic in Asian countries as well as Western countries. These results indicate that there is a need to improve the mental health of caregivers who are at risk for higher levels of parenting stress during the COVID-19 pandemic globally.

## Introduction

On March 11, 2020, the outbreak of the novel coronavirus disease 2019 (COVID-19) which began in Wuhan, China in December 2019 was classified by the World Health Organization (WHO) (World Health Organization, 2021) as a pandemic, a situation that has persisted for more than a year. Since the beginning of the pandemic, many schools worldwide were closed for an average of 3.5 months in 2020 (UNICEF, 2021). As a result of these prolonged school closures, children have often shown tendencies toward mood swings, anxiety, emotional problems, and behavioral and cognitive changes (Fegert et al., 2020; Francisco et al., 2020; Jiao et al., 2020; Tso et al., 2020; Tang et al., 2021). Further, the restrictions on daily life due to school closures and those placed on activities affect not only children’s mental health but also that of their caregivers (Cusinato et al., 2020).

Previously, we were the first to survey the impact of prolonged school closure due to the COVID-19 pandemic on the mental health of caregivers in Japan. Our survey of caregivers with children out of school revealed an increase in parenting stress compared to pre-pandemic levels. Further, qualitative analysis using textual mining of free descriptions regarding parenting stress during the pandemic indicated that spending a lot of time with their children was the primary stressor (Hiraoka and Tomoda, 2020). A high number of cumulative stressors in caregivers due to school closures and quarantine measures related to the pandemic has been shown to be associated with higher parenting stress, which indirectly becomes a potential risk factor for child maltreatment (Ramaswamy and Seshadri, 2020; Afrin and Zainuddin, 2021; Lee et al., 2021). Thus, several reports have indicated the importance of adequate mental health care for caregivers (Brown et al., 2020; Marchetti et al., 2020; Spinelli et al., 2020). As the pandemic shows no indications of ending soon, increased risk of parenting stress among caregivers, as described above, is an urgent issue that must be addressed in consideration of children’s healthy development and lifelong physical and mental health of parents and children.

From August to September 2020, shortly after we reported the findings of our previous survey conducted in Japan, a large-scale outbreak occurred in India, unlike any sudden rise in cases during a pandemic seen before in Asia. As a result, India became the country with the second highest number of COVID-19 infections worldwide after the United States; then, the tension about the spread of COVID-19 increased. Subsequently, the number of cases in Malaysia began to increase around September 2020, before dramatically increasing starting around December 2020 and continuing to grow (Supplementary Figure 1). Nevertheless, few epidemiological studies examining the influence of restrictions placed on activities during the pandemic, on the mental health of children and their caregivers have been conducted in Asian countries, except for China, as there had not yet been large-scale outbreaks such as those in Europe and the United States. However, the Indian government declared a state of emergency in March 2020, and imposed school closures and restrictions on activities for 7 months, which was twice as long as the global average. In Malaysia, a movement control order (MCO) was declared in March 2020. School closures and restrictions on activities have been repeatedly imposed since, to varying extents. Recently, a state of emergency was declared and continues to be in operation in Malaysia. Although the situation has been very serious, with many severe cases and deaths, the social situation of children and caregivers in these Asian countries seems to have been similar to that in Japan, Europe, and the United States.

In addition to biological vulnerabilities, such as psychiatric disorders, brain substrates, and genetic and epigenetic variations among caregivers themselves, psychological and social vulnerabilities such as poverty, single parenting, lack of social support, and the caregivers’ own childhood experiences of maltreatment can also be significant risk factors for engaging in child maltreatment in the context of situations where parenting stress is exceptionally high (Bowers and Yehuda, 2016; Shimada et al., 2018, 2019; Hiraoka and Nomura, 2019; Park et al., 2019; Cecil et al., 2020; Kuboshita et al., 2020; Hiraoka et al., 2021; Kasaba et al., 2021). One vital psychological and social risk factor for engaging in child maltreatment, regardless of country, ethnicity, or culture, is when caregivers themselves had adverse childhood experiences (ACE) (Felitti et al., 1998). It has been reported that the more adversity experienced in childhood, the higher the vulnerability to stress in adulthood (Albott et al., 2018). Even in the absence of unprecedented circumstances such as a pandemic, child-rearing often involves stress; thus, caregivers who experience more adversity in childhood may be at higher risk of developing child-rearing difficulties (Lange et al., 2019). Individuals who have experienced childhood adversity have been shown to exhibit mental vulnerability to the large-scale social change of COVID-19 (Guo et al., 2020). In addition to the ACE, a higher level of anxiety related to COVID-19 in caregivers may also be an additional contributing risk factor to increased parenting stress. However, since this high anxiety level may interact with caregivers’ ACE, it is also necessary to consider this structure when comprehensively investigating increases in parenting stress.

Therefore, the present study first aimed to investigate whether parenting stress among caregivers increased across Asia, as it did in Europe and the United States (Brown et al., 2020; Calvano et al., 2021; Lee et al., 2021), due to school closures and restrictions on activities during the COVID-19 pandemic. We chose India, which experienced an outbreak in August and September 2020 and had the world’s second highest number of infections, to represent South Asia; Malaysia, where the first rise in cases occurred after September 2020 and is still increasing, to represent Southeast Asia; and Japan, which was the target country of our longitudinal survey based on our previous study (Hiraoka and Tomoda, 2020), to represent East Asia. The United States, which has the world’s largest number of infections and is ethnically and culturally different from regions in Asia, was chosen as the reference country for comparison. The second aim of the present study was to examine whether there were any country-specific, cross-country, or cross-regional risk factors for increased parenting stress. Finally, the third aim was to clarify how childhood adversity is related to COVID-19 anxiety as a common risk factor for high parenting stress.

## Materials and Methods

### Data Collection

We collected data across the three Asian countries examined in this study—India, Malaysia, and Japan as representative cases for South, Southeast, and East Asia, respectively—in addition to the United States as a reference country. Participants from CrowdWorks (Japan) and Amazon’s Mechanical Turk (MTurk) (India and the United States) online worker pools provided consent for participation prior to beginning the survey on the impact of COVID-19 on parenting stress. We used pre-screening filters offered by the platforms to limit participant age (18-55 years; MTurk only), parenthood status, and location (Japan, India, and the United States). All information gathered was processed anonymously, and participants received an incentive of US$1.50. The survey was conducted entirely on the web *via* psyToolkit (Stoet, 2010, 2017) on any platform between September 28th to October 21st, 2020, approximately 1 week after the number of new cases rapidly increased in India. Responses from the same ID and those with the exact same free text, which were thought to be from the same individual, were excluded (39 in India, 4 in Japan, and 8 in the United States), as shown in Table 1. We simultaneously conducted an extra survey in Malaysia from September to November 2020, when the number of new cases had begun to gradually increase for the first time, as it would be valuable to prospectively capture the live dynamics of parenting stress from the beginning. Unlike the other countries where we used online worker pools, in Malaysia, participants were recruited as volunteers. All participants provided informed consent before the survey. The survey was conducted in a similar manner to those in India and the United States *via* psyToolkit (Stoet, 2010, 2017). The study protocol and all procedures were approved by the Ethics Committee of the University of Fukui, Japan (Assurance #FU-20200007).

**TABLE 1:** Demographics of the participants.

	India, *N* = 142^a^	Malaysia, *N* = 67^a^	Japan, *N* = 182^a^	United States, *N* = 203^a^
Age (year)	32.5 (6.8)	39.4 (4.7)	37.4 (6.4)	36.5 (8.7)
Female	51 (35.9)	48 (71.6)	145 (79.7)	120 (59.1)
Number of children	2.2 (3.4)	2.4 (1.1)	1.6 (0.6)	1.7 (1.3)
**Cohabitation**				
Children (single)	32 (22.5)	6 (10.7)	6 (3.4)	40 (19.7)
Children + spouse/partner	46 (32.4)	35 (62.5)	157 (88.2)	116 (57.1)
Children + spouse/partner + parent/others	64 (45.1)	15 (26.8)	15 (8.4)	47 (23.2)
**Race**				
Asian (including Indian)	139 (97.9)	53 (98.1)	182 (100.0)	7 (3.4)
Caucasian	0 (0.0)	0 (0.0)	0 (0.0)	166 (81.8)
Other	3 (2.1)	1 (1.9)	0 (0.0)	30 (14.8)
Ethnicity (Hispanic or latino/a)	NA	NA	NA	23 (11.3)
Education (> graduated college)	138 (97.2)	50 (74.6)	NA	175 (86.2)
**House hold income (per year)**				
<$30,000	89 (62.7)	40 (59.7)	25 (13.7)	34 (16.7)
30,000–$75,000	38 (26.8)	10 (14.9)	129 (70.9)	96 (47.3)
75,000<	15 (10.6)	17 (25.4)	28 (15.4)	73 (36.0)

*^a^Statistics presented: mean (SD); n (%). NA, not applicable.*

### Psychological Questionnaires

The Parenting Stress Index™, Third Edition, Short Form (Abidin, 1995) (PSI-SF) was used to measure parenting stress. Each item of the PSI-SF is rated on a five-point scale, ranging from 1 (strongly disagree) to 5 (strongly agree). We used three subscales which assess different types of parenting stress: Parental Distress (PD), the extent to which parents feel competent, restricted, conflicted, supported, and/or depressed in their role as a parent; Parent-Child Dysfunctional Interaction (P-CDI), the extent to which parents feel satisfied with their child and their interactions with them; and Difficult Child (DC), how a parent perceives their child to be, whether the child is easy or difficult to take care of, in addition to the total stress which is an indication of overall level of stress a person is feeling in their role as a parent. Participants were asked to complete the PSI-SF twice. First, the participants answered the PSI-SF without any particular instructions. Then, following completion, they were asked to complete the PSI-SF again, recalling what it was like before school closures and restrictions on activities. Such retrospective measuring methods have often been used when examining the health effects of COVID-19 (Gao and Scullin, 2020; Robillard et al., 2021).

In addition to the PSI-SF, we used the Coronavirus Anxiety Scale (CAS) to evaluate excessive concern and dysfunctional anxiety symptoms associated with the COVID-19 pandemic (Lee, 2020). Each item of the CAS is rated on a five-point scale, ranging from 0 (not at all) to 4 (nearly every day), based on experiences over the past 2 weeks. A CAS total score ≥ 9 indicates probable dysfunctional coronavirus-related anxiety (Lee, 2020). Elevated scores on a particular item may indicate problematic symptoms that could warrant further assessment and/or treatment.

Whether participants had ACE was determined based on their responses to the questions from the Centers for Disease Control (CDC)’s 2011 Behavioral Risk Factor Surveillance System (BRFSS) questionnaire, which contains 11 questions to measure three types of child abuse (physical, sexual, and emotional) and five types of household dysfunction (substance abuse, mental illness, domestic violence, incarceration/jail, and divorce/separation) (Merrick et al., 2018). Self-reported exposure to any single ACE category is counted as one point toward the final ACE score (range: 0–8).

### Qualitative Measurement of Parenting Stress

Participants were also asked to freely describe any parenting stress they were currently experiencing (“Are you experiencing parenting stress due to the spread of COVID-19? What kind of stress are you feeling as a result of school closures?”). Although this question was not compulsory, 89% of participants answered it. While participants from India, Malaysia, and the United States answered this question in English, Japanese participants answered it in Japanese. We analyzed the text data, which was translated into English *via* Google Translate for Japanese cases.

### Statistical Analysis

To investigate the effects on parenting stress from prolonged school closures and restraints on activities due to the pandemic in each country, a two-way mixed ANOVA was conducted. Considering the literatures on the COVID-19 pandemic highlights a greater impact on the female population (Lebel et al., 2020; Grumi et al., 2021; Malkawi et al., 2021), we also conducted a three-way mixed ANOVA to explore gender differences were existing. Additionally, to visualize the characteristics of the frequently reported words and the similarities or differences among countries, we conducted co-occurrence network analysis using KH-Coder (Higuchi, 2016, 2017). Words were extracted from free descriptions of parenting stress, and the top 60 words that occurred most frequently were extracted. In the co-occurrence network analysis, words that were unique to each country were linked to the circle of the country. If a word was similarly extracted across countries (e.g., stress or time), that word was linked to the respective country circle. Pearson correlation analyses were conducted between each outcome to examine any associations between PSI, CAS, and ACE. We conducted mediation analysis to assess whether the CAS mediated the link between ACE and PSI. The indirect effect was tested by bootstrapping confidence intervals using the lavaan package (Rosseel, 2012) of the R statistical software program (R Core Team, 2019). The model parameters were set to give bias-corrected 95% confidence intervals and to run 2,000 bootstrap resamples. Then, multi-group analysis was used to examine differences among countries in the path coefficients between PSI, CAS, and ACE. We compared the first (which allowed for the structural paths to vary across countries) and second models (which constrained the regression paths to remain the same for countries) to identify any country-related differences. In addition, to confirm if there were gender differences in the path coefficients among countries in the path analysis, we conducted multi-group analyses between men and women in each country. All statistical analyses were performed using R 3.6.1.

## Results

As shown in Table 2, a two-way mixed ANOVA for PSI-SF total scores revealed significant main effects for “country” [*F*(3, 526) = 32.7, *p* = 2.16E-19, η^2^ = 0.15] and “time” [*F*(1, 526) = 20.8, *p* = 6.43E-06, η^2^ = 0.002] in all the sub-scales and the total score. However, no significant interactions between “country” and “time” were observed for all cases [*F*(3, 526) = 0.9, *p* = 0.45, η^2^ = 0.0002]. Pairwise *t*-tests of PSI-SF total scores between each “country,” irrespective of the “time,” revealed India was the highest compared to the other countries (vs. Malaysia: *p* = 6.9E-22, vs. Japan: *p* = 1.2E-24, and vs. United States: *p* = 0.02; *p*-values were adjusted using the Bonferroni multiple correction method). All other combinations of the pairwise *t*-tests between each “country” were also significant (Malaysia vs. Japan: *p* = 9.55E- 3, Malaysia vs. United States: *p* = 0.02, and Japan vs. United States: *p* = 2.59E-16). No significant main effect for gender difference and interactions between gender and the other factors across the countries was found [*F*(1, 517) = 1.59, *p* = 0.21, η^2^ = 0.003]. Five participants were excluded from the analysis due to no gender assignment information available.

**TABLE 2:** The results of the psychological questionnaires.

	Time	India, *N* = 139	Malaysia, *N* = 39	Japan, *N* = 155	United States, *N* = 197	Statistics*
**PSI-SF**						
PD	Before	38.0 (13.6)	23.2 (7.7)	29.3 (9.2)	35.1 (12.6)	*F*(1, 526) = 18.7, *P* = 1.81E-05, η^2^ = 0.003
	After	39.3 (12.6)	25.2 (7.5)	30.5 (8.7)	36.6 (12.0)	
P-CDI	Before	35.2 (14.2)	20.6 (6.9)	22.7 (6.4)	32.0 (13.4)	*F*(1, 526) = 5.2, *P* = 0.023, η^2^ = 0.0005
	After	35.2 (13.0)	22.1 (7.0)	23.8 (6.4)	32.0 (13.0)	
DC	Before	35.2 (12.8)	22.5 (7.8)	27.3 (9.6)	33.7 (12.4)	*F*(1, 526) = 16.9, *P* = 4.63E-05, η^2^ = 0.002
	After	36.0 (12.1)	23.7 (7.1)	29.2 (9.0)	34.6 (12.1)	
Total	Before	108.5 (39.3)	66.3 (20.3)	79.3 (22.2)	100.8 (36.7)	*F*(1, 526) = 20.8, *P* = 6.43E-06, η^2^ = 0.002
	After	110.5 (35.7)	71.0 (19.5)	83.4 (21.3)	103.2 (34.8)	
CAS	–	7.5 (5.6)	0.6 (1.4)	2.3 (3.7)	5.3 (5.3)	
ACE	–	3.3 (2.1)	0.8 (1.1)	1.6 (1.6)	3.1 (2.2)	

Statistics presented: mean (SD), PSI-SF; The short form of the Parenting Stress Index, PD, Parental Distress; P-CDI, Parent-Child Dysfunctional Interaction; DC, Difficult Child; CAS, COVID-19 anxiety scale; ACE, Adverse childhood experience. *Two-way mixed ANOVA main effect of “Time.”

The results of the co-occurrence network analysis for open-ended statements regarding parenting stress are shown in Supplementary Figure 2. Commonly, “child” and “school,” which may be related to school closure, were reported as parenting stressors in each country. In addition, the word “time” was also found for all countries. While there were some positive comments, such as that school closures allowed children to spend more time at home and for parents to spend more time with their children, there were also several comments indicating that parents did not have time to relieve their own stress (e.g., “*I am stressed a lot more because my kids are ALWAYS here. Them being here all the time bring more chores, bills, and less me time. I never get privacy anymore just like when they were toddlers.*”). This suggests that it may be necessary to find ways for parents to secure time for themselves in the limited space of the home to maintain their mental health during the pandemic.

Pearson correlation analyses between the PSI-SF (including sub-scales), CAS, and ACE across all countries revealed they were robustly correlated with each other (Supplementary Figure 3). For example, total PSI-SF scores were significantly correlated with CAS scores (*r* = 0.61, *p* = 5.5E-59), and ACE (*r* = 0.53, *p* = 3.7E-42). All within-country combinations were also significant.

We designed the mediation model with CAS as a mediating variable, ACE as an explanatory variable, and PSI as an outcome variable. In this model, the paths from ACE to CAS (a) and from CAS to PSI (b) were significant (*a* = 1.05, SE = 0.09, *p* = 2E-16; *b* = 2.97, SE = 0.27, *p* = 2E-16), and the total effect was also significant (total effect = 8.13, SE = 0.56, *p* = 2E-16). There was a significant indirect effect [indirect effect = 3.11, SE = 0.38, 95% CI = (2.41, 3.88)]. Furthermore, the direct effect of ACE on PSI was until significant after addition of the mediator (direct effect = 5.02, SE = 0.58, *p* = 2E-16), which indicated CAS had a partial indirect effect on PSI.

**FIGURE 1:**
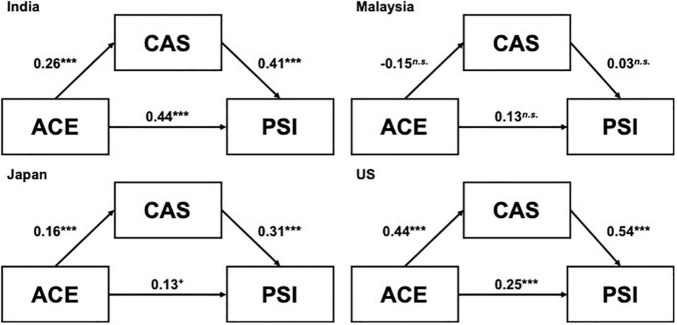
The cross-cultural mediating relationships between adverse childhood experiences, anxiety about COVID-19, and parenting stress. The paths represent unstandardized regression coefficients. ACE, Adverse childhood experience; CAS, Coronavirus Anxiety Scale; PSI, Parenting Stress Index (Total score). ***p < 0.001, *p < 0.05, ^+^*p* < 0.10.

Finally, we ran a multi-group analysis to examine whether the path coefficients differed significantly between countries (Figure 1). A model with no constraints on the path coefficients and a model with equality constraints were compared. The significant chi-square differences indicated that the regression coefficient differed by country [χ^2^(9) = 47.7, *p* = 2.9E-07]. The AIC of the former model (8530.8) was smaller than that of the latter model (8560.5), and the model without constraints was adopted. In both India and the United States, the coefficients between each variable were high, indicating that ACE was closely related to anxiety about COVID-19 and parenting stress. A similar relationship was found in Japan, but the path coefficients were not necessarily higher than India and United States, and ACE was not significantly associated with parenting stress. Finally, regarding Malaysia, the associations among each variable were not significant and the coefficients were smaller than the other countries. These results suggested that ACE may not necessarily be associated with anxiety and parenting stress in Malaysia. Or, there is another possibility that ACE may have been under-reported by participants due to the prevailing cultural norms in Malaysia. The multi-group analyses, in which a model with no constraints on the path coefficients and a model with equality constraints were compared between men and women in each country, showed no significant Chi-square differences indicating that the regression coefficient did not differ between gender [India: χ^2^(3) = 6.61, *p* = 0.09, Malaysia: χ^2^(3) = 3.52, *p* = 0.32, Japan: χ^2^(3) = 3.34, *p* = 0.34, and United States: χ^2^(3) = 6.41, *p* = 0.09].

## Discussion

This study examined the social and psychological risk factors contributing to increased parenting stress amidst the COVID-19 pandemic, across countries. The results showed that, as we expected, parenting stress increased under the COVID-19 pandemic in all the Asian countries and the United States compared to the pre-pandemic period. This study is the first to concurrently examine and compare parenting stress in three different Asian countries. We also found that there was originally a difference in PSI scores among countries regardless of the period, which has been discussed in detail later. However, India had the highest PSI scores, followed by the United States of the sample countries. The mediation analysis showed that the number of ACE was strongly associated with higher parenting stress, and that anxiety and fear about the pandemic itself mediated the effect.

When we conducted this online survey in September–October 2020, the United States and India were the first and second most infected countries globally, while Malaysia and Japan had relatively fewer cases than other countries where COVID-19 was prevalent. Nevertheless, both Malaysia and Japan showed an increase in parenting stress during the pandemic. Other studies conducted in Italy and Germany showed that parents experienced more parenting-related exhaustion due to social distancing as well as the closure of schools and child-care facilities, which manifested itself in increased parenting stress (Marchetti et al., 2020; Calvano et al., 2021). However, these studies were conducted in countries with high numbers of infections and deaths, unlike Malaysia and Japan. Therefore, the present study suggests that the cause of increased parenting stress is likely not only from factors directly related to the severity of the COVID-19 pandemic, such as the high number of infections and deaths but also other factors. Many Asian countries, including Malaysia and Japan, repeatedly declared a lockdown, MCO or state of emergency to combat the pandemic; schools were closed, activities were restricted, and many experienced social isolation (Nazif-Muñoz et al., 2021; Tang et al., 2021). It is to be noted that the social environment changed drastically due to unemployment and telecommuting (Lawson et al., 2020). The results of our textual analysis showed that the words “children,” “school,” and “time” were commonly reported as the specific stressors of the participants in every country. Although some participants viewed the situation in a positive light, reporting that they had “more family time since schools were closed and children spent more time at home,” many viewed it negatively, saying that “they had less free time and did not have time to relieve their stress.” The inflation of these inconveniences compared to the pre-pandemic era, and the maladaptation to new environmental changes, may have led to increased parenting stress. For example, Dickerson and Kemeny (2004) conducted a meta-analysis of the relationship between the characteristics of stressors and cortisol responses, and found that tasks containing uncontrollable elements were associated with robust cortisol reactivity (Dickerson and Kemeny, 2004). It is possible that in a situation where action is restricted worldwide, the physical symptoms of stress response, such as exhaustion, or changes in sleeping habits (Elhadi et al., 2021) are also seen, which may have led to an increase in parenting stress. However, there are some individual differences in tolerance to stressors. Individuals vulnerable to ambiguity are more stressed and rate their subjective well-being lower (Hancock and Mattick, 2020). The spread of COVID-19 can also be considered as an ambiguous situation, unlikely to be resolved immediately (Durodié, 2020), and the adaptation to this situation may be affected by individual differences. Further identification of factors may be necessary for the future.

We also found that childhood adversity as well as anxiety and fear about COVID-19 were risk factors for higher parenting stress during the pandemic. If high parenting stress is a risk factor for child maltreatment, our results support the well-known theory concerning the intergenerational cycle in survivors who were exposed to maltreatment during childhood that leads them to maltreat their child after becoming a parent (Lange et al., 2019; Uddin et al., 2020). In the mediation analysis, it was found that those with more ACE were more likely to have higher anxiety and fear about the COVID-19 pandemic. A model for predicting parenting stress has been suggested from childhood adversity, anxiety and fear about COVID-19. Numerous studies suggest that individuals who have experienced many childhood adversities, are vulnerable to hypothalamic pituitary adrenal (HPA) axis responses and a variety of other psychiatric disorders (Heim et al., 2000). Kalia et al. (2020) reported that individuals who were maltreated as children, but were not exposed to other social adversities such as poverty, were associated with fear of COVID-19, which resulted in higher anxiety. Moreover, given that a higher number of ACE is linked to greater susceptibility to parenting stress, this influence may occur with additional reinforcement in this unusual situation. The simultaneous multi-population analysis revealed that this mediation model was not completely common in every country. Cultural differences may exist across countries. Guo et al. (2020) studied maternal mental health in China, Italy, and Netherlands under the impact of the current pandemic and reported the protective effect of grandparenting support and higher number of children, on mental health symptoms of Chinese mothers, but not Italian and Dutch mothers. Protective/risk factors on maternal mental health may differ according to each country’s socio-cultural background. Thus, the relationship between ACE, anxiety and fear due to COVID-19, and parenting stress might be influenced by each country’s socio-cultural background, including history, culture, ideology, and values. India and the United States, where the influence of ACE was profound, were the regions with the most significant spread of infections globally during the study (October 2020), where parents were more likely to be anxious about COVID-19. Those who had ACE and were highly vulnerable to stress were more likely to be affected emotionally by their childhood adversity in situations where the infection rate had substantially increased such as India and the United States. It is possible that the link between the ACE and anxiety and fear in these countries appeared stronger than in Malaysia and Japan. It appears that the pandemic situation and lockdown will continue intermittently. Above all, when the infection rates rise, social support and mental health care would be required, especially for individuals with more ACE than usual.

Furthermore, our data indicated that the original level of parenting stress differed in each country, and India had the highest level. In some areas in India, the standard of living remains low (International Monetary Fund, 2021); thus, economic poverty may have been directly related to high parenting stress. Furthermore, insufficient mental health care systems (Sharma et al., 2007), labor shortages, high population densities that make it easier for infections to spread, and shortages and price surges of face masks and other protective equipment may also have contributed to the high levels of parenting stress (Haque et al., 2020). Moreover, it was noticed that many of the respondents in India were men (64%). Under the pandemic, men’s burden of housework and childcare may have increased. In contrast, several studies have reported that COVID-19 has increased the psychological burden on Indian women (Gopal et al., 2020; Malhi et al., 2021). Traditionally, there is a longstanding concept of “patriarchy” in certain parts of India, a family structure in which the father has absolute power and control over family members. Therefore, Indian women and children tend to internalize distress from an early age, based on the socially accepted notion that externalizing feelings is unacceptable (Levey et al., 2017). The extra stress caused by the unexpected pandemic added to the original oppressive stress may result in a higher level of parenting stress than in other countries. Parenting stress in the United States was the second-highest after India, probably because the United States has been the world’s leading country in terms of the number of infections and deaths for an extended period. This may lead to a high level of anxiety about the threat to life directly related to COVID-19 infection.

The present study has six major research limitations. First, it did not have a consistent online survey platform across countries. India and the United States used Amazon MTurk, Japan used CrowdWorks, and Malaysia did not use any online worker pool. As a result, there was a bias in the number of participants and their characteristics among the countries. Therefore, cautions should be taken when comparing our results from Malaysia with those of other countries. However, the online survey itself was effective as it allowed us to promptly collect a large amount of data in a short period because of the ever-changing situation due to the COVID-19 pandemic and the period of social distancing and self-isolation. Second, the sample size of the present study was relatively small than the other previous studies. We started the present study based on our previous study in Japan (Hiraoka and Tomoda, 2020), and thus we have tried to match its sample size for the other countries. Third, we did not match age and gender across the countries. There is a report that MTurk workers are predominantly male (Djellel et al., 2018). In this instance, the percentage of males was high in India and the United States. Fourth, the evaluation instrument was a self-administered questionnaire, leading to a bias toward socially desirable answers. Although there is a problem of accuracy, since online behavioral experiments are now available (Stoet, 2010, 2017), a more objective evaluation could have been made if such experiments were utilized. Fifth, the assessment of parenting stress before the pandemic was conducted using a retrospective response method. Although the reliability of retrospective response methods may be questioned as the data is limited to individual data, it is more consistent at the population level (Lena et al., 2020). Therefore, we did not use retrospective response methods in our analysis except when comparing before and after the pandemic. Longitudinal studies in particular, should be conducted prospectively. However, as we had to capture response promptly due to the unpredictable situation, this method was unavoidable. Finally, the timing of the survey may influence the outcomes. Parenting stress may fluctuate depending on the situation of the infections at the time.

## Conclusion

In the present study, we found that parenting stress increased in the three Asian countries and the United States during the COVID-19 pandemic. Higher number of ACE were strongly associated with higher parenting stress, an influential risk factor across countries. Anxiety and fear about the COVID-19 pandemic also mediated the effect. ACE may lead to vulnerable parenting and trigger stress responses which induce child maltreatment, which can be aggravated by a negative unprecedented situation. In addition to examining the caregivers’ current state, a retrospective assessment of past adversity experiences is warranted, because it can be expected to capture the risk of maltreatment more closely representative of the actual situation. A focus on ACE to provide more accurate support for parents and their evaluation should also be considered.

## Data Availability Statement

The raw data supporting the conclusions of this article will be made available by the authors, without undue reservation.

## Ethics Statement

The studies involving human participants were reviewed and approved by the Ethics Committee of the University of Fukui, Japan (Assurance #FU-20200007). The patients/participants provided their written informed consent to participate in this study.

## Author Contributions

SK, DH, and SN designed the study. SK conducted interviews with supervision from DH and SN. AA, SJ, NH, and AA-F collected the sample data from Malaysia. DH and SN completed all analysis and prepared tables and figures. SK wrote the main manuscript. Write up was completed by SK with critical revisions and supervision from DH and SN. All authors made substantial contributions to the analysis and data interpretation prior to agreeing upon key findings, conclusions, and approved the final manuscript.

## Conflict of Interest

The authors declare that the research was conducted in the absence of any commercial or financial relationships that could be construed as a potential conflict of interest.

## Publisher’s Note

All claims expressed in this article are solely those of the authors and do not necessarily represent those of their affiliated organizations, or those of the publisher, the editors and the reviewers. Any product that may be evaluated in this article, or claim that may be made by its manufacturer, is not guaranteed or endorsed by the publisher.
