# Supplementary figures and images for: Influence of the COVID-19 Pandemic on Parenting Stress Across Asian Countries: A Cross-National Study
Source: Front Psychol. 2021 Dec 21;12:782298. doi: 10.3389/fpsyg.2021.782298 (PMC8724041; doi:10.3389/fpsyg.2021.782298)

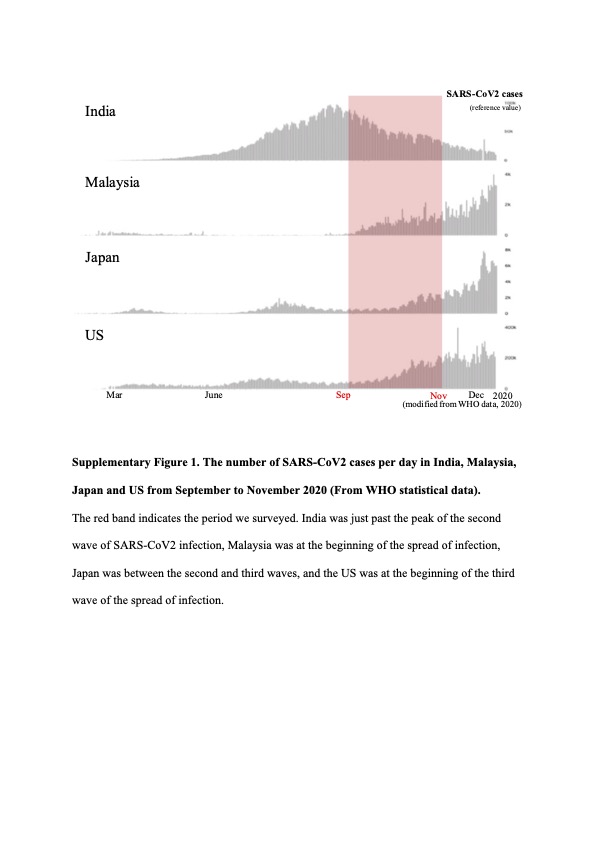

Supplement: Supplementary file 1 [file Image_1.JPEG]

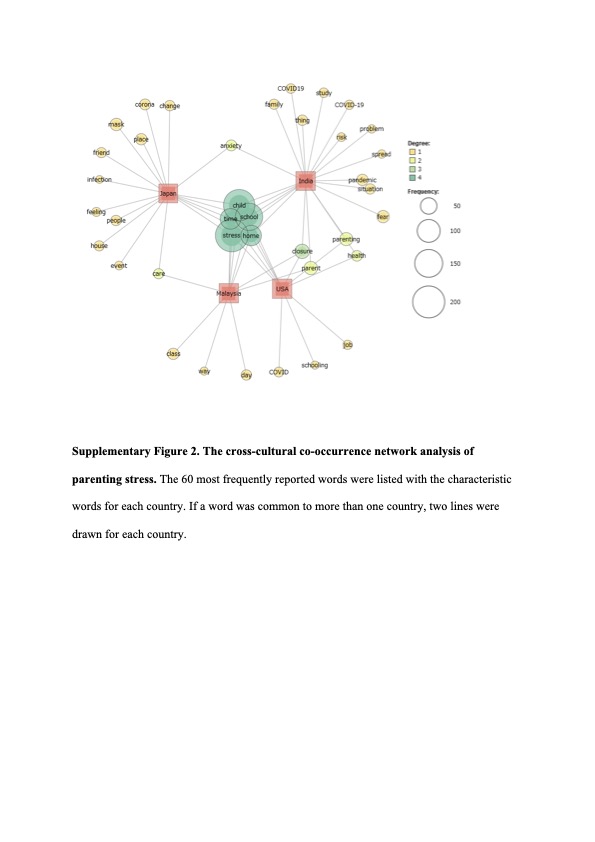

Supplement: Supplementary file 2 [file Image_2.JPEG]

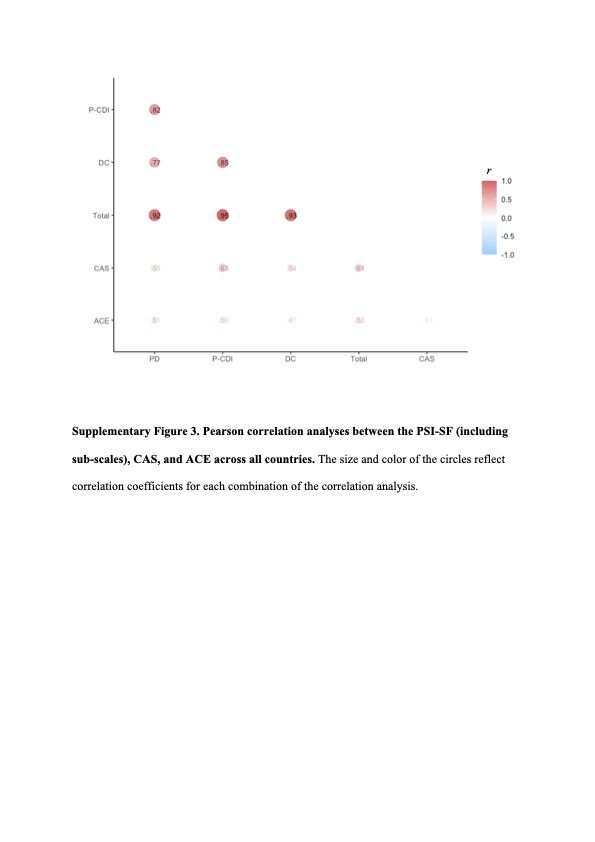

Supplement: Supplementary file 3 [file Image_3.JPEG]
